# The Detection of Bacteria in the Maxillary Sinus Secretion of Patients With Acute Rhinosinusitis Using an Electronic Nose: A Pilot Study

**DOI:** 10.1177/00034894231151301

**Published:** 2023-01-24

**Authors:** Jussi Virtanen, Antti Roine, Anton Kontunen, Markus Karjalainen, Jura Numminen, Niku Oksala, Markus Rautiainen, Ilkka Kivekäs

**Affiliations:** 1 Department of Otorhinolaryngology, Head and Neck Surgery, Tampere University Hospital, Tampere, Finland; 2 Faculty of Medicine and Health Technology, Tampere University, Tampere, Pirkanmaa, Finland; 3 Department of Surgery, Tampere University Hospital, Hatanpää Hospital, Tampere, Finland; 4 Olfactomics Ltd., Tampere, Finland; 5 Vascular Centre, Tampere University Hospital, Tampere, Finland

**Keywords:** maxillary sinusitis, electronic nose, ion mobility spectrometry

## Abstract

**Objective:**

Detecting bacteria as a causative pathogen of acute rhinosinusitis (ARS) is a challenging task. Electronic nose technology is a novel method for detecting volatile organic compounds (VOCs) that has also been studied in association with the detection of several diseases. The aim of this pilot study was to analyze maxillary sinus secretion with differential mobility spectrometry (DMS) and to determine whether the secretion demonstrates a different VOC profile when bacteria are present.

**Methods:**

Adult patients with ARS symptoms were examined. Maxillary sinus contents were aspirated for bacterial culture and DMS analysis. *k*-Nearest neighbor and linear discriminant analysis were used to classify samples as positive or negative, using bacterial cultures as a reference.

**Results:**

A total of 26 samples from 15 patients were obtained. After leave-one-out cross-validation, *k*-nearest neighbor produced accuracy of 85%, sensitivity of 67%, specificity of 94%, positive predictive value of 86%, and negative predictive value of 84%.

**Conclusions:**

The results of this pilot study suggest that bacterial positive and bacterial negative sinus secretion release different VOCs and that DMS has the potential to detect them. However, as the results are based on limited data, further conclusions cannot be made. DMS is a novel method in disease diagnostics and future studies should examine whether the method can detect bacterial ARS by analyzing exhaled air.

## Introduction

The diagnostics of acute rhinosinusitis (ARS) is a challenging task. Moreover, although the etiology of ARS is typically viral, bacterial prevalence is poorly defined.^1^ The most common bacteria reported are *Streptococcus pneumoniae, Haemophilus influenzae, Moraxella catarrhalis*, and *Staphylococcus aureus*.^2^ However, due to difficulties in differentiating the causative pathogen, antibiotics are often overprescribed.^3^ Thus, there is an urgent need for new innovative tools for the diagnosis of bacterial ARS.

Volatile organic compounds (VOCs) comprise a wide range of gaseous chemicals that are secreted in, for example, breath, urine, and saliva. Endogenous VOCs are the by-products of normal cell metabolism. In addition, they can also be directly released by microbes or as the result of an inflammatory response to infection. In contrast, exogenous VOCs are due to various external sources, such as smoking, diet, or air pollution.^4,5^ Detecting VOCs by olfaction has previously been studied in the diagnostics of diseases. For example, trained dogs were able to distinguish patients infected with SARS-CoV-2 from other virally infected patients by smelling nasopharyngeal and oropharyngeal swab samples with a sensitivity of 74% and a specificity of 95%.^6^

An electronic nose (eNose) attempts to mimic mammalian olfaction by analyzing VOCs from gas-phase mixtures. Traditional eNoses have sensors that react with gases and produce a signal pattern based on the chemical characteristics of the sample.^7^ Machine learning methods are then employed to discriminate samples that vary between subject groups.

Differential mobility spectrometry (DMS) provides analogous information on VOCs. The working principle behind DMS is the separation of ions based on their different mobility in high and low electric fields.^8^ For example, the technology is able to differentiate breast cancer tumors from benign samples with a sensitivity of 80% and a specificity of 90%.^9^ Moreover, an in vitro study revealed that DMS was able to discriminate the 4 most common reported ARS bacteria with an accuracy of 85%.^10^ Although the results are encouraging, they might be drastically different when samples are acquired ex vivo, as inflammatory response to infection and exogenous VOCs can interfere with the analysis of VOCs.^11^

The aim of this pilot study was to examine patients with ARS symptoms and to determine whether maxillary sinus secretions demonstrate different VOC profiles in DMS analysis when bacteria are present.

## Materials and Methods

### Participants

This prospective study was conducted at Tampere University Hospital, Tampere, Finland. The inclusion criteria were age ≥18 years and the presence of acute rhinosinusitis symptoms lasting less than 12 weeks as described in the European guidelines.^12^ Thus, patients were included if they had 2 or more symptoms, one of which being either nasal blockage/obstruction/congestion and/or nasal discharge. Additional symptoms were facial pressure or pain and/or reduction of or loss of smell. Exclusion criteria were smoking during the past 6 months, prior paranasal surgery, severe immunodeficiency, or any malignant disease treated in the previous 5 years.

All procedures performed in the study were in accordance with the 1964 Helsinki declaration and its later amendments or comparable ethical standards. The study was approved by the Ethics Committee of Tampere University Hospital (R16103). All patients provided written informed consent.

Information on the patients’ current rhinosinusitis symptoms and use of antibiotics 1 month prior to enrollment was obtained. A complete otorhinolaryngologic physical examination was then performed. Maxillary puncture and aspiration were conducted bilaterally unless a patient experienced only unilateral symptoms in which case the affected side was punctured.

As this was a pilot study, no sample size calculation was performed. The estimated number of 30 samples per group was based on our previous experience with DMS^10^ and a previous study.^13^

### Maxillary Puncture and Aspiration

First, a cream containing lidocaine 25 mg/g and prilocaine 25 mg/g was applied into the inferior meatus of the nasal cavity. In addition, a cotton-tipped aluminum swab containing adrenalin/epinephrine was placed into the inferior meatus for a few minutes for vasoconstriction and mucosal decongestion. The maxillary sinus puncture was then performed through the inferior meatus. The patient was placed horizontally, and after 30 seconds the maxillary sinus contents were aspirated with a 5 ml syringe. If no aspirates were received, we applied 2 ml of 0.9% sterile sodium chloride solution into the maxillary sinus and aspiration was repeated. If no pus was found in the aspirate, the syringe was discarded. The patient was then lifted back to the sitting position, and the sinus/sinuses irrigated with saline solution.

Approximately 0.5 to 1 ml of aspirate was then injected into an M40 Amies Agar Gel Transystem tube for bacterial culture. The remaining contents of the syringe were sealed with a cap, put in a sealed plastic bag, and stored in a fridge for later DMS analysis. Maximum storage time was set for 48 hours, but median storage time was 4 hours. After storing, the syringes were transported for DMS analysis. The time between transportation and analysis was between 15 and 40 minutes.

### Laboratory Analysis

The sinus aspirate samples were cultured on blood agar, chocolate agar, and fastidious anaerobic agar plates in aerobic and anaerobic conditions for 48 hours. Bacterial identification was performed according to standard procedures at the FIMLAB (Tampere, Finland) laboratories. The results of bacterial cultures were reported semi-quantitatively (slight growth, moderate growth, and heavy growth).

### DMS Analysis

**Figure 1.**
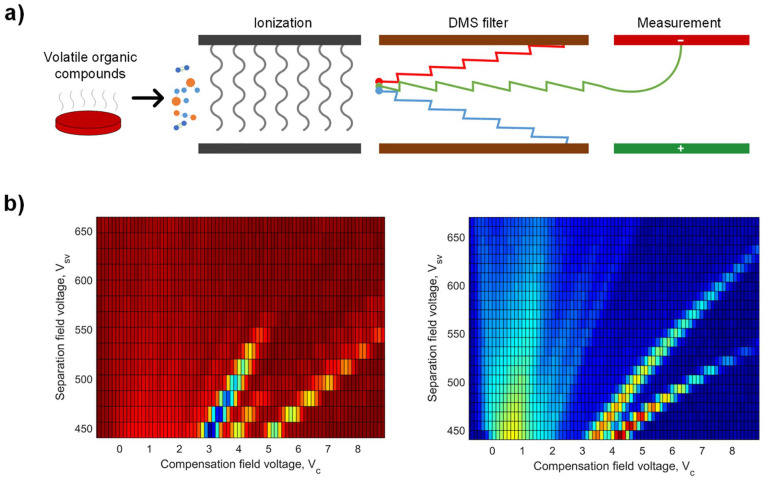
A schematic of the working principle of differential mobility spectrometry. (a) First, volatile organic compounds released by the sample are ionized and driven with a flow of air. The ions then enter a differential mobility spectrometry filter where they travel between 2 parallel electrodes and are exposed to intermittent high- and low-electric fields which causes the ions to oscillate. If the ions hit the electrodes, they are annihilated. A compensation voltage is introduced to allow the ions to pass to the detector to be measured. Ions colliding with the detector result in an electric current signal. (b) Output dispersion plots for negative (above) and positive (below) electrodes after measurement with different field voltages.

DMS ionizes molecules of the gaseous headspace and drives the ions into a drifting chamber formed by 2 electrodes. The mobility of an ion is affected by collisions with air molecules in the chamber which, in turn, depend on the mass, cross-section, and charge of the ions. In addition, DMS exposes the ions to intermittent high- and low-electric fields, which causes the ions to zig zag in the chamber, enhancing the separation power. Finally, the ions collide with the detector, resulting in an electric current signal. Tuning the voltage allows ion filtering and enhances the selectivity and sensitivity of the DMS. The output of a traditional DMS sensor is a 2D colormap, a so-called dispersion plot, where the *x*- and *y*-axes represent the electric field voltage values and the color represents the intensity of the current signal from the detector plate, that is, the number of ions that pass the filter stage with certain voltage values (Figure 1).

The DMS device used in this study was an ENVI-AMC™ (Environics Ltd., Mikkeli, Finland). The measurements were first conducted with purified air and tap water to set up a sensor baseline. The contents of the syringe were then injected onto an empty agar plate. Each plate was measured twice, producing 2 dispersion plots per sample. However, only 1 measurement was used in the data analysis. To reduce carry over from previous samples, tap water was measured after each sample plate. Each measurement lasted about 3 minutes.

Both negative and positive ions produced separate plots. The results were then stored in a cloud database (Olfactomics Ltd., Tampere, Finland) for later analysis.

### Data Analysis

MATLAB (The MathWorks, Natick, MA, USA) was used for data analysis. *k*-Nearest neighbor (*k*NN) and linear discriminant analysis (LDA) were used to classify samples as positive or negative. *k*NN and LDA are commonly used and rather simple supervised machine learning methods. In *k*NN, a measurement is assigned a class based on a distance measure to k training samples in the feature space. For example, if *k* is 2, the sample is classified based on its 2 nearest neighbors in the feature space, with priority given to the closest neighbor in case of a tie (ie, neighbors are of a different class). In LDA, the classification is based on a linear projection of the data features that maximizes the class separability. For cross-validation, the leave-one-out (LOOCV) method was used. Using this method, the classifier is built with all the samples except one, which is then used to test the classifier. The procedure is repeated as many times as there are samples, so that each sample is used once as a test sample. The estimate error of the classifier is the average of all the runs. Finally, the accuracy, sensitivity, specificity, and the negative and positive predictive values for the classifier were calculated. Thereafter, the Wilson score interval method was used to calculate 95% confidence intervals (CI).

## Results

A total of 26 samples from 15 patients were obtained. Nine (9/26, 35%) of the samples were culture positive and 17 (17/26, 65%) were culture negative. Of the positive cultures, 5 were *Streptococcus pneumoniae*, 1 *Haemophilus influenzae*, 1 *Streptococcus milleri*, 1 *Citrobacter koseri*, and 1 *Aggregatibacter aphrophilus*. Heavy bacterial growth was observed in all samples except for 1 sample, where the bacterial growth was slight (*S. pneumoniae*). This patient had heavy growth of the same bacteria in the contralateral sinus, so both findings were considered positive.

Nine of the 15 (60%) patients had received prior antibiotic treatment and 4 patients had positive bacterial culture in at least 1 sinus. Three of the 6 patients who had not received prior antibiotic treatment had positive bacterial culture.

After LOOCV, *k*NN (*k* = 2) produced accuracy of 85% (95% CI, 66-94) sensitivity of 67% (35-88), specificity of 94% (73-99), positive predictive value of 86% (49-97), and negative predictive value of 84% (62-94; Table 1). LDA produced accuracy of 73% (54-86), sensitivity of 56% (27-81), specificity of 82% (59-94), positive predictive value of 63% (31-86), and negative predictive value of 78% (55-91).

**Table 1.** Confusion Matrix Presenting the Results of *k*-Nearest Neighbor When *k* = 2.

	Estimated class	
	Bacteria +	Bacteria −
Bacterial culture +	6 (TP)	3 (FN)
Bacterial culture −	1 (FP)	16 (TN)

Abbreviations: FN, false negative; FP, false positive; TN, true negative; TP, true positive.

## Discussion

In the present study, we evaluated the concept of the analysis of VOCs released from maxillary sinus secretion using an eNose based on DMS. The results suggest there might be a difference in VOC profile between bacterial positive and bacteria negative samples. These VOCs are most likely also present in the nasal cavity, and thus support the hypothesis that they could be differentiated by an analysis of the VOCs in the nasal air using DMS.

This view is supported by a study by Thaler and Hanson^13^ in which exhaled breath was collected using a modified nasal continuous positive airway pressure (CPAP) mask attached to an eNose. In their second experiment, the authors recruited 34 patients who were suspected of having bacterial rhinosinusitis based on clinical criteria. Endoscopically guided bacterial cultures were obtained from the nasal cavity for reference. The results were compared to 34 healthy controls. After LOOCV, the eNose could diagnose infected patients with an accuracy of 72%. The study did not, however, compare infected patients to each other, and therefore it is unknown whether patients with different bacteria would have had different results.

As demonstrated in previous studies, eNose technology can differentiate bacteria. Swabs containing common upper respiratory tract pathogens taken from bacterial plates could be differentiated from each other using an eNose.^14^ Furthermore, an in vitro study revealed that DMS can distinguish the 4 most common acute rhinosinusitis bacteria with an accuracy of 92% using *k*NN and LOOCV.^10^ The analysis of VOCs, however, gets more complicated when performed with ex vivo samples, as these samples also contain endogenous and exogenous VOCs.^11^ As a result, the findings of potential biomarkers in in vitro studies have not been reproduced in clinical studies.^15^ Indeed, the VOCs-based diagnostic method is still in its infancy and warrants further research.

Unfortunately, our results are limited by the small number of samples as demonstrated by the wide CI. Our aim was to collect more samples, but the COVID-19 pandemic complicated the study as maxillary punctures were no longer performed in our clinic at the beginning of the pandemic to avoid the spread of the virus in aerosols. As a result, the decision was taken to end the study.

In total, 35% of samples were culture positive. This finding was probably the result of prior antibiotic consumption. Nevertheless, the typical acute rhinosinusitis bacteria *Streptococcus pneumoniae* and *Haemophilus influenzae*, in addition to 2 rarer ones, were found in the culture samples. Furthermore, the results raise the question as to whether antibiotic consumption had an effect on the VOCs in the sample. Of course, patients with no prior antibiotic consumption should ideally be compared to those patients who had used antibiotics to see whether there is a difference.

The limited number of bacterial positive samples affected the construction of the classifier and its ability to classify samples correctly. This was seen in the sensitivity, which only reached 67% at best. Another limitation of this study is that we were unable to assess whether DMS can distinguish specific bacteria in the sinus secretion. To have done so, we would have needed multiple samples of each bacterium to build a classifier to recognize them all. Furthermore, it remains unclear whether DMS can distinguish a true infection from colonization or detect polymicrobial infection. It has been shown that a combination of pathogens alters the signal pattern of the eNose.^16^ In addition, DMS does not at present provide information about antibiotic sensitivity, such as that provided by a standard bacterial culture. Interestingly, Saviauk et al^17^ revealed that an eNose based on ion mobility spectrometry could differentiate methicillin-sensitive and methicillin-resistant Staphylococcus aureus with accuracy of 91%.^17^

The performed LOOCV reduces overoptimistic results and provides confidence that the results are reproducible. However, due to the small sample size in the present study, it was not possible to perform external validation with training and test sets. This form of validation would give a better estimate of the prediction error of the classifier when applied to a new population.^18^

As the detection of bacterial ARS is clinically challenging, it is difficult to avoid the unnecessary use of antibiotics. Therefore, innovative methods are warranted. Using eNose technology, the detection of diseases is possible as has been shown in the diagnostics of cancer and respiratory diseases.^19,20^ Further studies should evaluate whether the performance of DMS to detect bacterial ARS improves when combined to clinical diagnostic data including symptoms, clinical examination, and laboratory testing. For example, secretion in the posterior pharynx, moderate or profuse secretion in the nasal passage or facial tenderness may indicate a bacterial etiology when symptoms have lasted 9 to 10 days.^21^ Therefore, the sensitivity and specificity of DMS could potentially increase when these findings are also considered. Furthermore, an analysis of the breath air of patients with ARS should be explored with DMS, as this would be a non-invasive sampling method. If it becomes possible to detect bacterial ARS in such a way, DMS will be a valuable additional tool for diagnosing ARS.

## Conclusion

The results of this pilot study suggest that bacterial positive and bacterial negative sinus secretions release different VOCs and that DMS technology has the potential to detect them. The results are, however, based on limited data and further conclusions cannot therefore be made. DMS is a novel method in disease diagnostics and future studies should examine whether the method can detect bacterial ARS rhinosinusitis by analyzing exhaled air.
